# Mitigation Techniques of Membranes’ Biofouling in Bioelectrochemical Cells (BEC Cells): Recent Advances

**DOI:** 10.3390/membranes15110332

**Published:** 2025-11-01

**Authors:** Shatha Alyazouri, Muhammad Tawalbeh, Amani Al-Othman

**Affiliations:** 1 Sustainable & Renewable Energy Engineering Department, University of Sharjah, Sharjah P.O. Box 27272, United Arab Emirates; u22102266@sharjah.ac.ae (S.A.); mtawalbeh@sharjah.ac.ae (M.T.); 2 Sustainable Energy & Power Systems Research Centre, RISE, University of Sharjah, Sharjah P.O. Box 27272, United Arab Emirates; 3 Department of Chemical and Biological Engineering, American University of Sharjah, Sharjah P.O. Box 26666, United Arab Emirates; 4 Energy, Water and Sustainable Environment Research Center, American University of Sharjah, Sharjah P.O. Box 26666, United Arab Emirates

**Keywords:** microbial cells, membrane biofouling, mitigation techniques, electro-assisted technology, hydrophilicity, membrane modification, ceramic membranes

## Abstract

Biofouling remains a critical challenge in bioelectrochemical cells (BECs), hindering their efficiency and performance. This research article reviews advances in biofouling mitigation techniques within BEC systems during the period from 2019 to 2025, focusing on membrane modifications and electro-assisted membrane technologies. Through comprehensive analysis, it is revealed that Nafion alternatives, including ceramic membranes and recycled nonwoven fabrics like polypropylene, have emerged as significant contenders due to their combination of low cost and high performance. Additionally, the incorporation of silver, zeolite, and graphene oxide onto membranes has demonstrated efficacy in mitigating biofouling under laboratory conditions. Furthermore, the application of direct current electric fields has shown potential as a chemical-free preventative measure against biofouling in BECs. However, challenges related to long-term stability, scalability, and cost-effectiveness must be addressed for widespread adoption.

## 1. Introduction

Environmental concerns, fossil fuel depletion, energy demand growth, and the diversity of renewable resources and technologies have led to promoting renewable energy [1,2]. However, the intermittent and unpredictable nature of renewables, which results in low load factors of just 22% and 12% for wind and solar power, respectively, presents a significant challenge to grid stability [3,4]. This challenge of intermittency, however, has also been a powerful catalyst for innovation, accelerating the large-scale development and utilization of energy storage and energy conversion devices [5]. These technologies allow for the storage of excess renewable energy, which can then be dispatched to the grid when solar and wind resources are unavailable, thereby ensuring a reliable power supply and reducing the need for fossil fuel backup. On the other hand, biomass represents a renewable and abundant resource for energy production, distinguished by its widespread availability and accessibility across various regions, making it a viable local resource. Additionally, it is compatible with a diverse range of energy conversion technologies [6].

Bioelectrochemical (BEC) systems have emerged as highly efficient and sustainable technologies for converting carbon-rich waste into valuable products, including bioenergy, nutrients, metals, and chemicals [7]. This is accomplished by utilizing live microorganisms in electrochemical processes, including electricity production [8], bioelectrochemical synthesis [9], wastewater treatment [10,11], and hydrogen and methane production [12].

A typical BEC cell comprises two electrodes, specifically the anode and cathode, which are interconnected via an external circuit. Internally, these electrodes are separated by an ion exchange membrane, facilitating ion transport between the anode and cathode while maintaining distinct electrochemical environments [13]. The BEC system can be classified into a microbial fuel cell (MFC), microbial electrolysis cell (MEC), microbial desalination cell (MDC), and microbial electrosynthesis system (MES) [14].

In a biocatalyzed or microbial electrolysis cell, electrochemically active microorganisms are utilized to transform dissolved organic material into bicarbonate, protons, and electrons, ultimately yielding hydrogen. These microorganisms release electrons to an electrode surface, either through direct contact or with the assistance of (excreted) redox mediators, thereby generating a current [15]. Connecting the biological anode to a proton-reducing cathode via a power supply enables the direct conversion of dissolved organic material into hydrogen. Unlike photo fermentation [16], this renders the process independent of reactor surface area, enhancing economic viability.

Theoretically, hydrogen production via MEC of acetate requires only 0.14 V of applied voltage. However, practical implementation suggests that more than 0.14 V may be necessary [17,18]. This discrepancy arises from several factors, like electrochemically active microorganisms utilizing a portion of the available energy for their own growth and maintenance, releasing electrons at a higher potential than the equilibrium. Other losses within the cell, such as ohmic resistance and overpotentials, are expected to influence the required applied voltage. In contrast, water electrolysis requires practically an applied voltage of 1.6 V [19].

This entire process occurs within an electrochemical cell, where the oxidation of dissolved organic material and proton reduction are partitioned into two chambers, usually separated by a membrane [1,20]. As the power supply drives the released electrons from the anode to the cathode, an equal number of protons traverse the membrane. Upon arrival at the cathode, protons and electrons combine, resulting in the production of pure hydrogen gas [21]. Therefore, ensuring a sufficient flow of ions between the anode and cathode is crucial to maintain charge equilibrium. Mainly, the purpose of the membrane is to provide a physical barrier to avoid short-circuiting and the crossover of random ions and substrates between the anode and cathode sides [22]. In many electrochemical systems, the benchmark material for such membranes is Nafion, a perfluorosulfonic acid (PFSA) ionomer, which exhibits a unique nanostructure, high proton conductivity, and exceptional chemical stability. Its architecture, comprising a hydrophobic PTFE backbone and hydrophilic sulfonic acid-terminated side chains, drives nanoscale phase separation, forming interconnected ionic domains essential for proton transport. Table 1 summarizes the key properties resulting from this structure and their significance:

However, the use of Nafion in BEC systems faces two significant challenges: its high cost, driven by a complex and energy-intensive synthesis process, and its susceptibility to the same operational issue that plagues other membranes [28].

**Figure 1:**
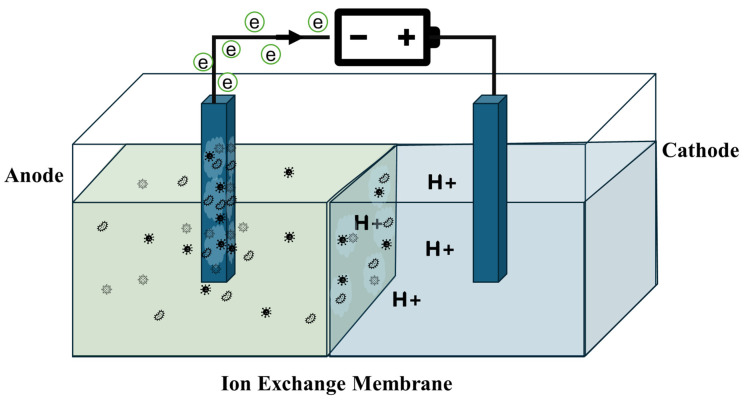
Schematic of a microbial electrolysis cell showing anode–cathode compartments separated by an ion exchange membrane. Various symbols in the anode represent various microbes used.

The direct contact between organic and inorganic substances and the membrane gives rise to biofouling [29], as can be seen in Figure 1, presenting a significant challenge in membrane operations.

**Figure 2:**
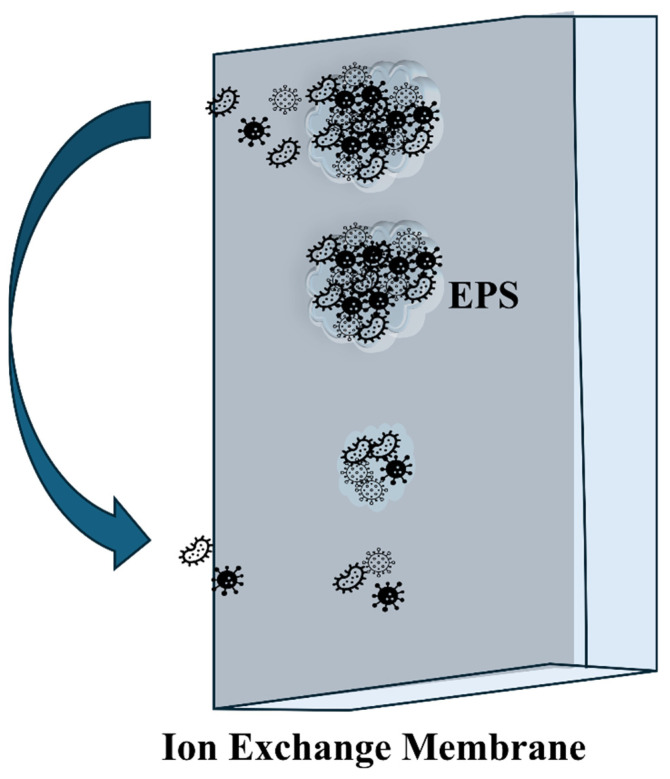
Stages of biofilm formation on a membrane surface.

Four main types of fouling can limit the performance of microbial electrolysis cells (MECs): colloidal fouling, organic fouling, scaling, and biofouling, with biofouling being the most predominant [30]. Biofouling begins when microorganisms interact with the membrane surface through various physical and electrostatic forces. The extent of these interactions depends on both microbial characteristics and membrane properties, including surface charge, wettability, and surface morphology [31]. This is followed by biochemical interactions between bacterial cells and the nutrients or materials present around the membrane surface, which promote further microbial growth. Subsequently, microorganisms release a substance known as extracellular polymeric substances (EPS), which facilitates the formation of the biofilm [32]. Several studies have been conducted to control and affect the production of EPS, like changing the operating conditions and adding materials to the wastewater [33,34,35,36,37]. The stages of biofilm formation are demonstrated in Figure 2. The biofilm continues to grow by releasing additional EPS and attracting more microorganisms. This process continues until the biofilm is disrupted, releasing microorganisms and initiating the cycle once again. Biofilm development, along with many essential biological processes within bacterial communities, is often regulated by a cell density-dependent mechanism known as quorum sensing (QS) [38]. Disrupting bacterial communication systems, such as QS, has been suggested as a promising tactic for managing bacterial biofilm formation, a critical aspect of biofouling development [39,40,41,42].

The nature of biofouling in BECs is distinct from other membrane-based processes like reverse osmosis (RO) or membrane bioreactors (MBR). In RO/MBR, fouling primarily impacts hydraulic permeability and filtration efficiency. In contrast, BEC membrane fouling directly degrades electrochemical function, where even a thin biofilm can cause significant ohmic losses and disrupt the delicate proton and charge balances essential for current generation and synthesis reactions. This fundamental difference underscores the need for antifouling strategies specifically designed for the unique electrochemical and biological environment of BECs.

**Figure 3:**
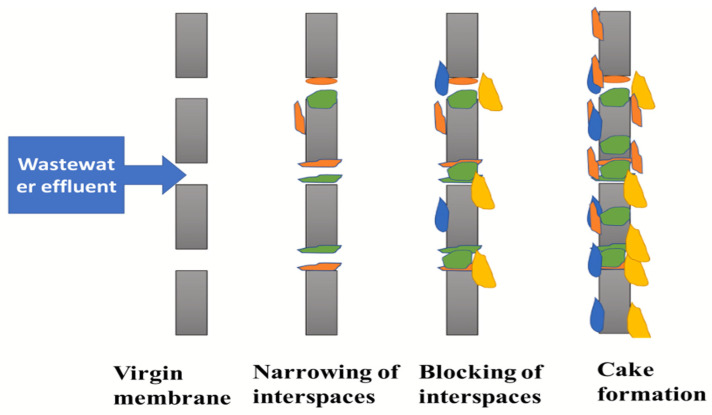
Membrane pore blockage by biofouling formation [30].

The biofilm formation extends to grow on other parts of the MEC, like the membrane and the cathode, resulting in a reduction in power density by 32% [43] and power generation by 50% [44], respectively. This breaks the carbon-carbon bridge at the cathode, blocking the oxygen reduction reaction. Moreover, the biofilm formation affects the membrane proton conductivity and ion transfer mechanisms due to the reduced pore size and blockage of the membrane pores, as illustrated in Figure 3 [30].

Fouling can be mitigated via two routes: module selection and system operation, and membrane selection and design [45,46]. Module selection and system operation mitigate biofouling by promoting mass transfer away from the membrane, reducing foulant buildup [47,48]. Configurations like crossflow increase shear at the membrane surface, which prevents particles from adhering. Key diffusion processes, such as shear-induced diffusion, inertial lift, and lateral migration, will keep particles in motion, reducing biofouling risk. Design features like turbulent flow, mixing inserts, and techniques like air sparging and backwashing further disrupt foulant accumulation [49]. Operational strategies, including pretreatment and adjustments to pH and ionic composition, also help by lowering foulant concentrations and reducing their affinity for the membrane.

Both module configuration and membrane design ultimately aim to minimize the physicochemical drivers of microbial adhesion at the membrane–solution interface. While operational strategies enhance hydrodynamic shear and mass transfer to suppress particle deposition, the intrinsic surface characteristics of the membrane, such as hydrophilicity, surface charge, and roughness, govern the subsequent likelihood of irreversible attachment. Therefore, effective antibiofouling design requires an integrated approach that couples module-level optimization (flow dynamics, mixing intensity) with membrane-level engineering of surface chemistry and topography to achieve synergistic suppression of biofilm initiation.

**Figure 4:**
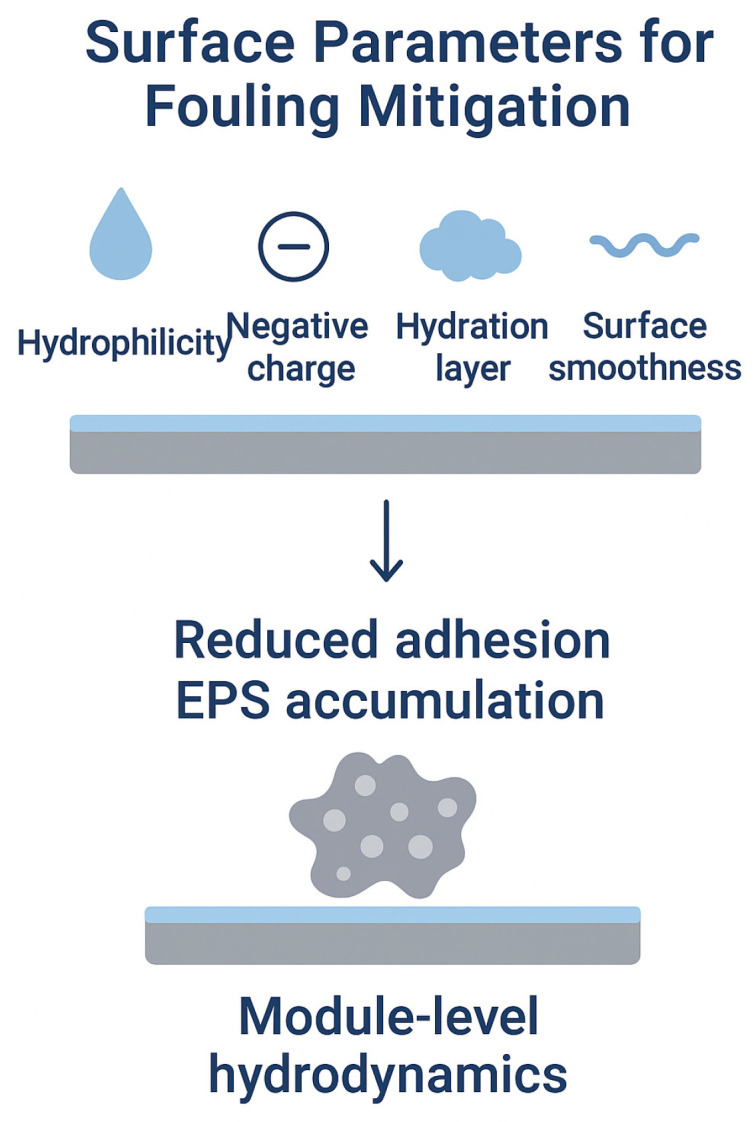
Primary anti-fouling mechanisms rely on a combination of hydrophilicity, negative surface charge, and surface steric/osmotic repulsion.

Membrane selection minimizes biofouling by reducing surface affinity to feed components, focusing on hydrophilicity, negative charge surface, surface smoothness and hydrogen bond acceptance [50,51,52]; refer to Figure 4. Hydrophilic surfaces, as noted by Kang and Cao [53], bind water and lower foulant adsorption, while smooth surfaces and hydrophilic polymer chains resist fouling through steric and osmotic repulsion. Materials like nylon and cellulose are naturally hydrophilic, whereas polysulfone and polyethersulfone (PES) can be modified for hydrophilicity, offering low fouling potential for ultrafiltration [54]. For materials that are hydrophobic, such as polyvinylidene fluoride (PVDF) and polytetrafluoroethylene (PTFE), hydrophilic versions are available, though pilot testing is often needed [55]. When ideal membranes are not commercially available, modification methods like polymer blending, interfacial polymerization, and graft polymerization are employed to optimize the membrane’s surface properties [45].

Multiple studies reviewed mitigation techniques of membrane biofouling in BEC technologies [30,56,57,58,59]. Jadhav et al. [30] reviewed biofouling formation, bioelectrochemical cell (BEC) configurations to mitigate biofouling, and advances up to 2021, including membrane surface modifications and the use of biocides. Pasternak et al. [56] provided a comprehensive review of methods to identify microbial communities, biofilm structure, and anti-fouling strategies. Nasruddin and Abu Bakar [57] investigated the impact of biofouling on several industries, like wastewater treatment, the marine industry, and biofuel cells (BFCs). Biofouling can be prevented using QS disruption, quaternary ammonium compounds (QACs), graphene oxide (GO), metal oxides, and silver ions. Koók et al. [58] reported the methods of membrane biofouling characterization in BEC, in addition to some techniques of membrane biofouling, like the use of ionic liquid-based membranes and the modification of commercial membranes. The most recent comprehensive review article that reported the advances in biofouling mitigation was published in 2022 by Desmond et al. [59] covering literature up to the first quarter of that year. However, the field has since experienced a rapid and substantial acceleration in research. Our analysis of the literature confirms this surge, with 58 highly relevant publications (approximately 31.3% of our total references) emerging between mid-2022 and late 2025. This significant body of new work drives the need for an updated analysis.

This review aims to address this critical gap by providing an updated and focused analysis of these advancements, specifically covering the period from 2022 (Q2) to 2025 (Q4).

However, the field of biofouling mitigation has seen rapid and substantial developments since then. This review aims to address this critical gap by providing an updated and focused analysis of recent advancements in biofouling mitigation techniques across BEC systems, specifically covering the period from 2019 to 2025. This work uniquely emphasizes membrane material modifications and the role of electro-assisted membranes in biofouling control. We delve into emerging strategies such as the integration of novel nanomaterials, the development of cost-effective Nafion alternatives, and the application of direct current electric fields as a chemical-free preventative measure. By exploring these solutions, this review aims to identify effective strategies to reduce biofilm formation, enhance membrane performance, and optimize overall cell efficiency, while also highlighting the very latest research trends in the field.

## 2. Methodology

This article conducts a systematic review to identify the most recent advances in antibiofouling mechanisms of bioelectrochemical cells (BECs). The review followed established systematic review protocols to ensure transparency and reproducibility.

### 2.1. Data Sources and Search Strategy

**Figure 5:**
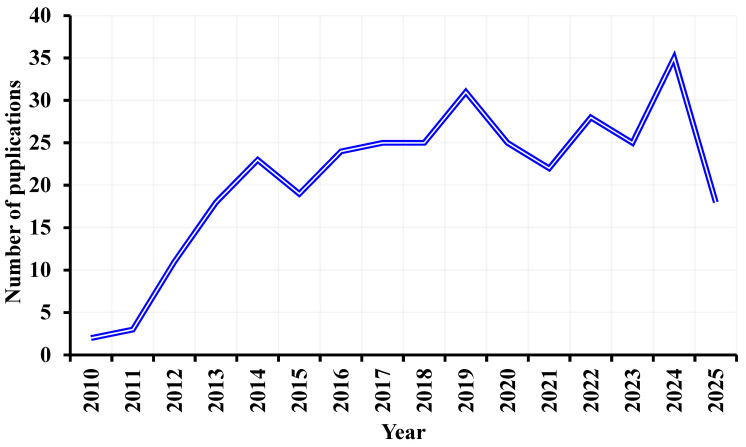
Number of publications addressing biofouling in BEC cells.

Academic studies were gathered from Google Scholar and Scopus. The search focused on articles published between 2019 and 2025. Keywords were selected to address the research objectives, including relevant synonyms, and combined using Boolean operators. The final search string used in Scopus was:

“((microbial electrolysis cell) OR (microbial fuel cell) OR (bioelectrochemical cells) OR (BES) OR (BEC) OR (biocatalyzed)) AND ((biofouling) OR (fouling) OR (anti biofouling) OR (biological fouling)) AND ((Membrane) OR (separator))”. Figure 5 illustrates a significant increase in the number of publications from 2010 to 2025, reflecting the growing interest in this topic.

### 2.2. Inclusion and Exclusion Criteria

○ Inclusion Criteria:
  ▪ Peer-reviewed journal articles focusing on antibiofouling mechanisms in BECs.
  ▪ Studies published in English between 2019–2025.
  ▪ Experimental or modeling studies reporting fouling mitigation strategies of membranes or separators in BECs.
○ Exclusion Criteria:
  ▪ Studies addressing cathodic biofouling exclusively.
  ▪ Articles focusing solely on novel BEC configurations or electrolyte modifications without membrane fouling analysis.
  ▪ Review articles, book chapters, conference abstracts, or non-peer-reviewed studies

### 2.3. Screening and Selection Process

The initial search retrieved 69 articles from Google Scholar. However, many of these studies deviated from the main topic. After removing redundant articles, irrelevant studies, book chapters, and review articles, 35 articles remained for screening.

The search in Scopus retrieved 102 articles, of which 50 articles remained after excluding book chapters, review articles, and irrelevant studies.

Matching the results from both databases identified 43 unique articles that met all inclusion criteria and were included for full-text review and qualitative synthesis.

## 3. Results and Discussion

### 3.1. Limitations of Conventional Ion-Exchange Membranes

**Figure 6:**
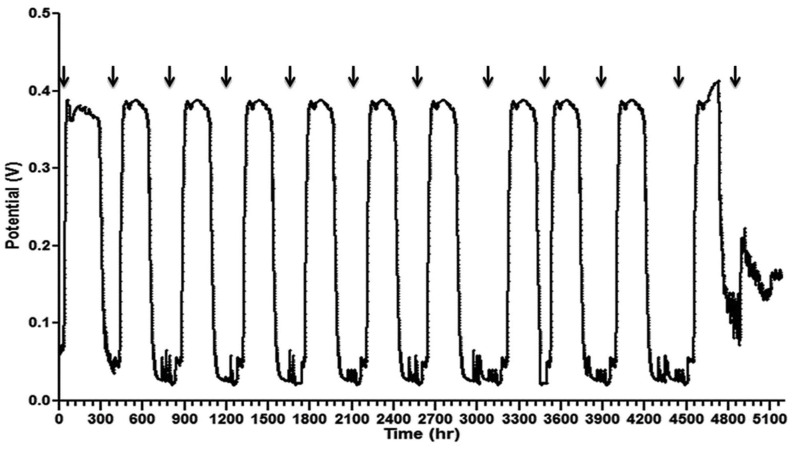
Deterioration in power generation at the Nafion membrane due to biofouling was observed after six months of operation without cleaning [63].

In a two-chamber configuration, membranes or other separators are used to divide the electrochemical cell into anode and cathode half-cells. For a long time, Nafion was the primary reference material for two-chamber MFCs [60]. However, the use of Nafion and other ion exchange membranes in MFCs has revealed significant limitations, such as low proton selectivity, substantial gas and substrate crossover, high production costs, and membrane fouling [61]. Both chemical fouling and biofouling remain critical issues that have not yet been fully explored in terms of their impact on MFC performance [62]. Flimban et al. [63] investigated biofouling on Nafion using scanning electron microscopy (SEM); biofouling was prominently visible, covering nearly the entire membrane on the cathode side. The combined biofouling on both the anodic and cathodic sides contributed to the observed decrease in power generation by 37%, which occurred after approximately six months of stable, repeatable output in the MFC, as shown in Figure 6.

San-Martín et al. [64] studied the mechanical, chemical and electrochemical properties of five different commercial cation exchange membranes (CEM) and found that Nafion and CMI membranes showed significant roughness increases, indicating higher fouling susceptibility, while Zirfon, FKB, and FKE had slower fouling progression; refer to 2. These findings highlight Nafion’s limitations in fouling resistance despite its widespread use. A promising approach is the modification of the commercial Nafion membrane by using NPs [65]. Sigwadi et al. [66] prepared a nanocomposite membrane by modifying Nafion with sulfated zirconia oxide (SZrO_2_) and SZrO_2_-(NH_3_SO_4_), the modified Nafion membranes offered enhanced hydrophilicity with contact angles of (80–68°) and hence improved anti-biofouling properties. Table 2 shows a comparative performance of Nafion and alternative CEMs in MFCs [62,63].

### 3.2. Alternative Commercial and Low-Cost Membranes

Various commercial and low-cost membranes were tested in the literature for their antifouling properties [67,68]. San-Martín et al. [67] fed Two-Chamber MEC with pig slurry in a fed-batch mode for 103 days to assess CMI 7000 membrane deterioration. It was found that the ion exchange capacity deteriorated the most at the first and the sixth membranes, with a deterioration rate of 22%. Haupt et al. [68] membrane-electrode assembly with an antibacterial Fumion^®^ FFFA-3 membrane on the gas diffusion electrode showed minimal, easily removable biofilm formation. Among antifouling methods tested, UV radiation was the most effective in preventing biofilm growth on gas diffusion electrodes (GDEs).

Recently, low-cost alternative membranes, including ceramic, non-woven cloth, and PVDF, have been investigated [69,70]. The use of recycled non-woven cloth to manufacture membranes will have a positive impact on the environment and the economic competitiveness of the membrane preparation process. Pasternak et al. [71] manufacture polypropylene nonwoven fabric (PP80) for manufacturing two polymer/ceramic membranes, namely, PP-373 and PP-468. The experiments, PP-373, maintained the lowest contact angle over time. The 373-CMP membrane decomposition ended at a similar weight when compared to the unused control 373-CMP, indicating a reduction in biofouling over time. Luthfiana et al. [72] investigated the development of sustainable antifouling ultrafiltration membranes from recycled cigarette filter waste modified with tannic acid and FeCl_3_ coatings. Antifouling performance was enhanced due to the use of tannic acid and FeCl_3_ coatings, which formed a hydrophilic and protective surface layer. Resulting in higher flux recovery (76% compared to 60% for unmodified membrane) and with reduced irreversible fouling but increased reversible fouling, enabling easier cleaning and longer membrane lifespan.

Recent research emphasizes modifying PVDF-based membranes to enhance hydrophilicity and biofouling resistance. Sun et al. [73] developed a novel PVDF-co-HFP membrane enhanced with PP13-TFSI ionic liquid and chlorosulfonic acid (P-I&S). This modification increased membrane hydrophilicity from a contact angle of 91° to 62°, enhanced proton conductivity from 0.025 S/cm to 0.087 S/cm, and improved biofouling resistance, resulting in a 2.1-fold increase in current production (from 0.48 mA/cm^2^ to 1.01 mA/cm^2^) and a 3.2-fold increase in power density compared to unmodified membranes. The MFCs incorporating P-I&S membranes also demonstrated prolonged cycling stability over 120 noncycles, highlighting their potential for cost-effective and efficient BEC applications. Further demonstrating the potential of synthetic polymer composites, a novel SPEEK/SSA membrane functionalized with phosphomolybdic acid-decorated silica (SPMA) nanofillers was investigated by Solomon et al. [74]. The optimal membrane (5% SPMA) demonstrated a high proton conductivity of 3.75 × 10^−2^ S cm^−1^, a maximum power density of 196.6 mW m^−2^, and exhibited significant anti-biofouling properties with up to 67.52% biofilm inhibition, outperforming Nafion-117.

### 3.3. Biodegradable and Ceramic Membrane Alternatives

Exploring other low-cost materials, a novel chemically modified Cellophane separator with polydimethylsiloxane (PDMS) presents a compelling cost-performance compromise for MECs using real waste streams. Colantoni et al. [75] discovered that while unmodified Cellophane is extremely low-cost (€5–10/m^2^) and effectively prevents pH imbalance like commercial AEMs, it biodegrades completely within one month. The PDMS modification successfully doubles its operational lifespan to two months at a still-low cost (€50–70/m^2^, or almost 10% of an AEM) and maintains stable anode pH, though it exhibits higher organic acid crossover (0.2 mg m^−2^ s^−1^) than ion-exchange membranes. This positions PDMS-Cellophane as a viable, biodegradable separator for short-to-medium term applications where ultra-low cost is critical.

**Figure 7:**
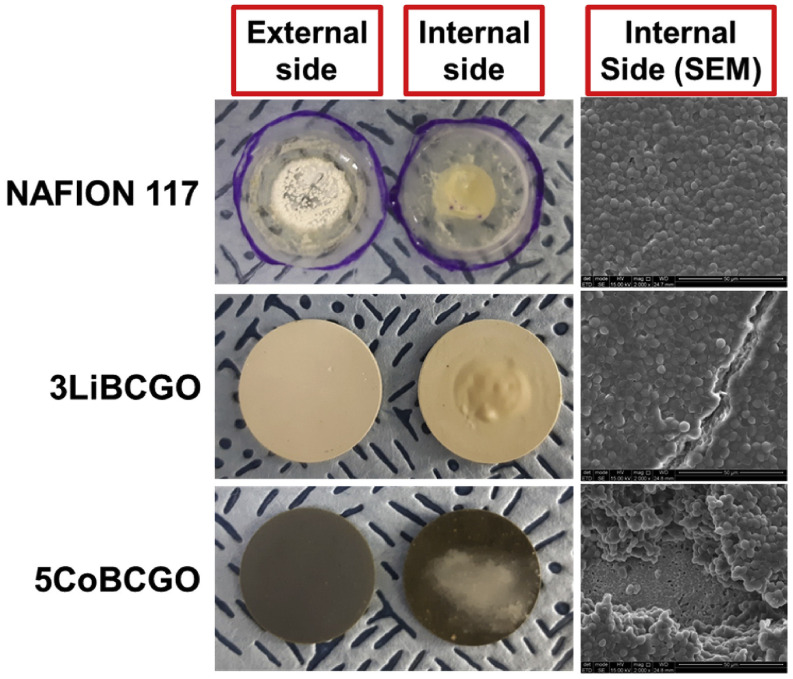
Photographs and SEM images of biofilm growth on BCGO, 3LiBCGO, and 5CoBCGO ceramic membranes after 24 h operation (SEM at 15 kV, 5000× magnification). The thinnest biofilm layer was observed on 5CoBCGO, demonstrating superior antifouling performance [77].

The operational concept of ceramic membranes is based on water permeating, diffusing, and evaporating through the microporous structure of the ceramic diaphragm. Ceramic membranes offer inherent hydrophilicity and adjustable surface charge, resisting fouling [76]. Frattini et al. [77] synthesized a ceramic membrane by incorporating borium, cerium, and gablinioum oxide powders, which were subsequently co-doped once with lithium and once with cobalt, resulting in three membrane samples, namely, BCGO, 3LiBCGO, and 5CoBCGO. Ceramic membranes prevent yeast cells from being transported with ions and water through the membrane. While the 3LiBCGO membrane exhibited a tenfold increase in water flux and the highest absolute biofilm mass (8.0 mg) in YPD + yeast medium, the 5CoBCGO membrane demonstrated superior overall performance, matching Nafion’s controlled YPD + yeast permeance (1.86 × 10^−3^ vs. 2.33 × 10^−3^ mol^2^·m^−2^·s^−1^·kJ^−1^) while achieving the lowest absolute biofilm accumulation (3.2 mg) and a 25% thinner biofilm structure than Nafion, as illustrated in Figure 7.

### 3.4. Ionic Liquid and Ionogel Membranes

Ionic liquid (IL) membranes have emerged as a promising class due to their enhanced charge and mass transfer and improved electrical performance [78,79]. The ILs are salts in the liquid state that have several desirable characteristics, such as high thermal and electrochemical stability, and high ionic conductivity under anhydrous conditions [80]. The incorporation of ILs into polymers enhances their stability and performance, as seen in bio-cellulose derived from bacteria, which offers a low-cost alternative [81]. When ILs are encapsulated within a polymer network, forming an ionogel, they exhibit excellent mechanical and electrochemical properties [82]. Szakács et al. [83] tested bio-cellulose-[BMIM][Cl] ionogel membrane in an acetate-fed MFC. Despite exhibiting a 4.5 times higher ionic conductivity (1.37 × 10^−4^ vs. 3.04 × 10^−5^ S/m) and a 16% lower oxygen crossover (1.38 × 10^−4^ vs. 1.61 × 10^−4^ cm/s) than Nafion 115, the cellulose-ionogel membrane’s performance was ultimately compromised by two critical flaws. Its substrate crossover was 13.7 times higher (1.17 × 10^−4^ vs. 8.52 × 10^−6^ cm/s), which promoted severe cathodic biofouling and led to worse operational metrics, including lower current density and Coulombic efficiency alongside a higher internal resistance. Furthermore, the membrane was found to be biodegradable, with its structure compromised by cellulolytic bacteria like Clostridium termitidis, which made up 23.2% of the anode-side biofilm, leading to its structural failure and rendering it inferior to the stable Nafion 115 in long-term MFC applications.

This challenge can be effectively overcome by selecting ILs with inherent antimicrobial properties, such as those containing quaternary ammonium groups. This principle is successfully demonstrated in the development of a low-cost NH_4_I-doped PVA membrane incorporating 4-ethyl-4-methylmorpholiniumbromide as the ionic liquid by Tomar et al. [84]. In contrast to the previous example, this strategic material choice yielded a membrane with a peak power density of 7.98 W/m^3^, a high ionic conductivity of 2.4 × 10^−2^ S/cm, and most notably, a reduction in surface biofilm biomass by over 60%.

### 3.5. Physicochemical Determinants of Antifouling Behavior

Mitigating microorganism adhesion on membrane surfaces involves modifying the membrane’s characteristics. This can prevent adhesion altogether or reduce it significantly. Through research, multiple characteristics of the membrane were found to be crucial to prevent biofouling formation, including hydrophilicity [71,85,86,87], negative surface charge [88,89], smoothness of the surface, and the antibacterial activity of the membrane composition [85,86,87].

The hydrophobic/hydrophilic characteristics of membranes are typically assessed through the contact angle measurement between the membrane surface and the surrounding liquid [90]. Conventionally, MFC/MEC membranes are crafted from materials exhibiting hydrophobic properties. However, the hydrophobic nature of these membranes can foster the adhesion of pollutants due to the absence of hydrogen bonds between water molecules and the membrane surface, consequently increasing entropy and facilitating the adsorption of microorganisms onto the membrane surface. Conversely, hydrophilicity forms a protective water barrier layer that impedes bacterial attachment.

One promising method of enhancing the hydrophilicity of a hydrophobic membrane is through doping a hydrophilic substance into the membrane matrix [91]. Lu et al. [10] modified thin film composite (TFC) polyamide FO membrane using Ag NPs, which led to enhanced hydrophilicity, increased negative surface charge, and antibacterial properties, resulting in biofouling prevention. Nagar et al. [86] increased the hydrophilicity of polyvinylchloride (PVC) matrix by doping hydrophilic Zeolite 4A. As a result, a minimal bacterial attachment occurred on the membrane surface, attributed to their antibacterial properties and the high hydrophilicity of PVC and Zeolite 4A.

### 3.6. Biopolymer-Based Membranes

Building on the strategy of enhancing membranes with hydrophilic additives, researchers have increasingly turned to chitosan as a sustainable biopolymer matrix for developing advanced separators. As a naturally abundant, biodegradable polymer rich in amino and hydroxyl functional groups, chitosan offers inherent hydrophilicity, biocompatibility, and antimicrobial properties that make it particularly suitable for BEC applications. This potential is demonstrated in recent studies exploring chitosan-based composites: Pupiales et al. [92] developed a ZIF-8/chitosan composite that achieved exceptional ionic conductivity (0.099 S/cm) and remarkable antifouling performance, showing only 25.88% surface roughness increase compared to Nafion’s 179.48%. Mendes et al. [93] designed, fabricated, and tested photocatalyst-enhanced chitosan membranes to determine whether Er/Al/ZnO improves ionic conductivity and resistance to fouling and biofouling compared to unmodified and commercial membranes. The incorporation of Er/Al/ZnO into chitosan membranes markedly enhanced performance, surface resistance dropped from 11.03 to 6.84 Ω·cm^2^ (MQ), 10.08 to 5.15 Ω·cm^2^ (MQ1), and 8.07 to 5.06 Ω·cm^2^ (MQ2), and biofouling resistance improved to 8.06 Ω·cm^2^.

Moreover, the use of carbon products like GO, rGO, functionalized GO (FGO), or sulphonated GO (SGO) was shown to enhance the hydrophilicity, mechanical strength, proton conductivity, and negative zeta potential of the membrane. Ben et al. [87] incorporated AgGO-GO and GO on the Sulfonated Poly(ether ether Ketone) (SPEEK) membranes. The GO enhances the mechanical structure of the polymeric membrane as well as the proton conductivity. Ag-Go is an antibacterial material. AgGO-GO-SPEEK exhibits the minimum internal resistance, 23.83%, due to the GO hydrophilicity and Ag antibacterial activity. Xu et al. [94] incorporated a minimal 0.025 wt% of GO into a thin (7–12 µm) active layer, and the novel membrane drastically improved hydrophilicity (contact angle 47.29° vs. 75.9°), demonstrated superior anti-fouling performance (83% vs. 52% flux recovery), and offered excellent antibacterial properties. These laboratory-scale results are promising; however, the long-term stability of GO-based coatings and the potential for nanomaterial leaching in continuous-flow systems require further investigation at a larger scale.

### 3.7. Metal Nanoparticles and Quaternary Ammonium Compounds

**Figure 8:**
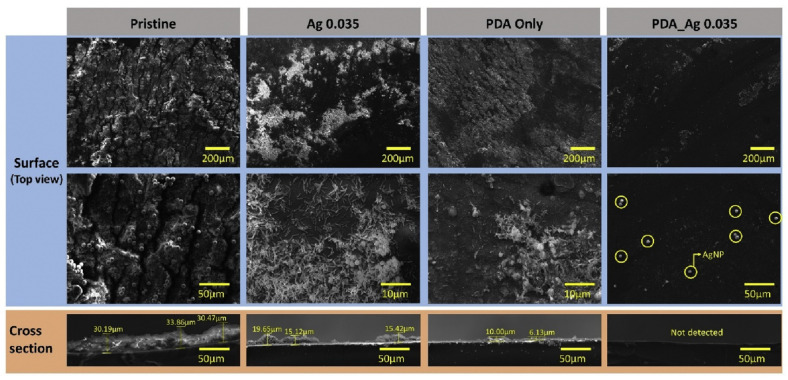
EM images comparing biofouling on pristine and modified SPAES/PIN-PE membranes with PDA and AgNP coatings. Imaging performed at 10 kV, 2000× magnification. The PDA-Ag membrane exhibited reduced microbial adhesion, confirming synergistic antibacterial and hydrophilic effects [97].

The distinctive characteristics of silver, including its chemical stability, conductivity, and antimicrobial properties, have directed significant interest towards its use [95]. Moreover, the abundant availability of silver and its effectiveness even in small quantities underscore its potential in mitigating biofouling. Silver’s capability lies in its ability to deactivate membrane cell wall enzymes by binding through thiol groups, catalyzing disulfide bond formation, and modifying protein structures [96]. This process ultimately leads to the inactivation of enzymes crucial for respiration, further justifying silver’s efficacy against biofouling. Park et al. [97] modified SPAES/PIN-PEM via surface functionalization with AgNP and PDA; multiple coating types and orders were investigated to overcome the problem of silver particles releasing from the membrane. The results are illustrated in Figure 8 [97].

The study has shown a biofouling reduction of 80.74% compared to pristine PEM. Contact angle of PDA-Ag 0.036 was 37.41°. And hydrogen production was maintained over time by a reduction of 31.88%. In addition, Kugarajah et al. [98] investigated multiple concentrations of silver incorporated with SPEEK, like SPEEK +2.5 wt.% Ag, SPEEK +5 wt.% Ag, SPEEK +7.5 wt.% Ag and SPEEK +10 wt.% Ag. It was noticed that the presence of biofilm formation was reduced. The addition of silver increased power density by mitigating biofouling over time. Even though silver is hydrophobic, SPEEK +7.5 wt.% Ag had a contact angle of 58.26°, which is higher than pristine SPEEK by only 2.2°. While these findings highlight the potential of silver nanoparticles, their economic viability for large-scale membrane fabrication and the environmental impact of silver leaching, which could promote antimicrobial resistance or cause ecotoxicity, present significant hurdles for real-world application.

In addition to metal nanoparticles like Ag NP, quaternary ammonium compounds (QACs) have emerged as a promising antimicrobial approach. QACs are particularly effective because their permanent positive charge enables strong electrostatic interactions with negatively charged bacterial cell membranes, leading to membrane destabilization, leakage of intracellular contents, and ultimately cell death [99]. Unlike other antimicrobial agents, QACs maintain their cationic nature across a wide pH range, ensuring stable and durable antibacterial performance. Moreover, when immobilized onto membrane surfaces, QACs can provide long-term bactericidal functionality without significant leaching, making them highly attractive for water treatment applications where both fouling resistance and microbial control are critical [100]. Li et al. [101] enhanced PVDF ultrafiltration membranes by surface-modifying them with dopamine-assisted co-deposition of zwitterionic (sulfobetaine-type MPC or carboxybetaine-type CBMA) and cationic (methacryloyloxyethyltrimethyl ammonium chloride, DMC) block copolymers, creating membranes that simultaneously resist protein adhesion and exhibit complete antibacterial activity. The best hybrid membrane (PDA/p(DCD)-1-PVDF) achieved BSA rejection >97%, flux recovery 84.78% (vs. 43.12% in pristine), FDR as low as 9.57%, and 100% antibacterial efficiency against *E. coli* and *S. aureus*.

However, recent studies emphasize the importance of balancing antibacterial performance with environmental safety. Although immobilization reduces leaching, trace silver release and QAC desorption may still occur, potentially contributing to antimicrobial resistance in wastewater systems [102]. To mitigate environmental risk, silver release from modified membranes must comply with drinking water standards, such as the U.S. Environmental Protection Agency’s secondary maximum contaminant level of 0.10 mg/L for silver [103]. Furthermore, supporting the sustainable deployment of these materials, end-of-life handling of Ag/QAC-modified membranes should move beyond disposal and include recycling to convert them into nanofiltration or ultrafiltration membranes, thereby preventing plastic waste and supporting a circular economy [104]. Table 3 displays biofouling mitigation studies with membrane modification.

### 3.8. Electro-Assisted Mitigation Methods

Recent findings have demonstrated that the application of a direct electric field during membrane operation efficiently deters charged microorganisms from attaching to the membrane surface, reducing the microorganism attachment to the membrane and the reduce the need for chemicals to control the fouling [110], consequently alleviating biofouling, refer to Table 4. This is attributed to the combined effects of electrostatic repulsion and electrocoagulation [111]. Increased aeration can also impact the directional motion of charged particles when subjected to a micro-electric field, thereby reducing the effectiveness of the electric field in mitigating membrane fouling. The balance between aeration levels and biofouling control is crucial for maintaining optimal membrane performance. Employing an electric field offers several advantages over conventional methods: as a non-chemical approach, it prevents the introduction of additional contaminants into the permeate, preserving water quality in the case of water treatment. Moreover, it supports continuous membrane operation without disruption. A primary limitation, however, is the energy consumption associated with maintaining the electric field, which impacts the net energy balance and economic feasibility of BEC systems. Additionally, electric field parameters, such as strength, frequency, and mode (continuous or pulsed), can be modified as required for enhancing fouling mitigation efficiency.

The antifouling efficacy of electro-assisted membranes is not merely a result of “non-chemical repulsion” but can be attributed to several well-defined physical and electrochemical mechanisms that occur simultaneously when an electric field is applied. The primary mechanisms include electrophoresis, electroosmosis, and in situ electrochemical oxidation [112]. Electrophoresis is likely the dominant mechanism for repelling microbial cells and other charged foulants. When a negative potential is applied to the cathode membrane, it generates a strong electrostatic repulsive force against the typically negatively charged bacteria and extracellular polymeric substances (EPS) in the solution. The velocity of this repulsive motion ($u_{ep}$) can be described by the Helmholtz–Smoluchowski equation (Equation (1)) [113]:

(1) $$u_{ep}=\frac{\varepsilon_{r}\varepsilon_{0}\zeta E}{\mu }$$

where $\varepsilon_{r}\varepsilon_{0}$ is the permittivity of the solution, ζ is the zeta potential of the foulant, E is the electric field strength, and μ is the dynamic viscosity. This equation quantitatively shows that the repulsion force is directly proportional to both the foulant’s surface charge and the strength of the applied field, explaining why higher currents often lead to better fouling control.

Electroosmosis, conversely, involves the movement of the bulk fluid. The applied potential induces a net flow of water through the membrane pores from the anode to the cathode. This electroosmotic flow acts in opposition to the permeate flow, effectively creating a counter-current that sweeps foulants away from the membrane surface and hinders their adhesion [114]. Furthermore, the applied electric field can drive in situ electrochemical reactions. For instance, water electrolysis at the electrodes can generate microbubbles (like H_2_ at the cathode) that physically scour the membrane surface, while the formation of reactive oxygen species (ROS) like hydrogen peroxide (H_2_O_2_) and hydroxyl radicals (•OH) at the anode provides a potent, localized biocidal effect that disrupts biofilm formation.

Hou et al. [115] investigated the effect of running a DC electric field on the biofouling formation while maintaining a constant aeration level of 1.5 L/m of MFC-MBR. It was found that biofouling can be avoided by the action of electrophoresis, electrostatic repulsive force, and electrochemical reaction. Moreover, Liu et al. [116] used a conductively modified AnMBR as the anode to study the effect of self-generated current (up to 0.44 mA at 10 Ω resistance) on biofouling mitigation, concluding that the current reduced membrane fouling by 59–82% for proteins and 66–83% for polysaccharides, while enriching electroactive bacteria. Anis et al. [117] developed an electro-ceramic self-cleaning membrane using nano-zeolite and CNS with PVDF for biofouling control in water treatment. Through periodic electrolysis, gas bubbles form on the membrane, sweeping away foulants and boosting flux by 21% after the first cycle and 17–23% in subsequent cycles. The composite’s antibacterial properties are enhanced by nano-zeolite (nano-Y) and CNS, achieving 82.3% yeast rejection and 67.1% SA rejection. The hydrophilic surface also aids quick regeneration, preventing reduced electrolytic efficiency due to bubble retention. This method provides effective, sustained biofouling mitigation. The incorporation of electric membranes into an anaerobic forward osmosis membrane bioreactor [118] and anaerobic membrane bioreactor [119,120,121] enhances antifouling performance by generating electrochemical repulsion, reducing organic buildup and sludge accumulation, preventing pollutant adhesion, and promoting biofilm detachment, ultimately extending operational efficiency and maintaining steadier flux over time. Sapireddy et al. [120] varied the specific cathode surface area (SCSA) of a dual-function cathode in an anaerobic electrochemical membrane bioreactor (AnEMBR) and tested its effect on biofouling. The larger size 8 m^2^/m^3^ effectively minimized biofouling due to its higher surface area, increased gas bubble production, and efficient dynamics of smaller bubbles.

The energy cost of this process is an essential factor to be considered; however, it remains in its early stages, with limited and inconsistent data across studies. Indirect costs, such as the manufacturing, replacement, and cleaning of membranes and electrodes, as well as direct costs for hydraulic power and electric field generation, are important determinants of costs [122,123]. Multiple studies reported energy consumption. Chiu [122] estimated 0.02 kWh/m^3^ for pumping and 1.70 kWh/m^3^ for the electric field, whereas Huotari et al. [123] reported 110 kW/m^2^ for pumping and 0.13 kW/m^2^ for the electric field. In addition, Bowen et al. [124] showed 2 kWh/m^3^ for pumping and 0.036 to 6.9 kWh/m^3^ for electric field operation. Conventional membrane cleaning costs, which include chemicals, backwashing, heating, and waste treatment, can range from 2.2 to 50.3% of operational costs [125]. Table 4 presents the biofouling mitigation by using an electric current.

**Table 4:** Biofouling mitigation by using electric current.

Type of the Cell	Membrane Type	Main Idea	Biofouling Capabilities	Ref.
**MBR**	DC electric field membrane	The use of electric fields to repel the bacteria; hence, there is less biofilm formation, accumulation, and blocking.	Lower zeta potential (−16.9 mV), soluble microbial product (SMP) decline (8.61–4.28 mg/L), and LB-EPS reduction (4.29–2.48 mg/g MLSS).	[115]
**MFC**	AnMBR (conductive membranes)	AnMBR was used as the anode to study the effect of passing a self-generated current on biofouling mitigation.	SMP, LB-EPS, and TB-EPS reductions: R-10 membrane exhibited 82.01%, 66.35%, and 72.57% lower polysaccharide content and 59.47%, 38.97%, and 80.42% lower protein concentrations compared to R-0 membrane.	[116]
**Cross-flow filtration and electrolysis setup**	Nano zeolite/CNS with PVDF as a binder.	Developing an electro-ceramic membrane made from nano-zeolite and evaluating its antifouling performance.	Periodic electrolysis cleaning increased flux by 21% after the first cycle, with subsequent cycles showing additional increases of 23%, 22%, and 17%.	[117]
**AnOMEBR**	Thin film nanocomposite FO membrane with nanocarbons.	Developing an electro-assisted anaerobic forward osmosis membrane bioreactor (AnOMEBR) to treat wastewater and reduce membrane fouling using a conductive FO membrane as both the separation unit and cathode.	SMP reduced by 26%, PN/PS ratio reduced by 15%, and steady flux decline (10.88–2.41 LMH in 134 h) compared to AnOMBR (9.60–1.15 LMH in 72 h).	[118]
**AnEMBR**	Carbon nanotube hollow fiber membranes 16 (CNTs-HFMs)	Electro-assisted Anaerobic Electrochemical Membrane Bioreactor (AnEMBR) using CNTs-HFMs to improve antifouling performance.	Lower transmembrane pressure (TMP) (0.35 bar), reduced EPS adhesion, minimized gel layer formation, and inhibited pollutant penetration.	[119]
**Single-chamber (AnEMBR)**	Nickel-based hollow fiber membrane (Ni-HFM)	Investigating biofouling control by varying the cathode surface-to-cathode area (SCSA).	The 8 m^2^/m^3^ AnEMBR effectively reduced biofouling.	[120]
**Single-chambered Membrane Bio-Electro-Reactor (MBER)**	Carbon fiber cloth composite membrane anode (PVDF/PVP-based)	Integrating electrooxidation with a conductive membrane anode to enhance ammonia removal and biofouling resistance.	1.4 V anode potential generated free chlorine, reducing PVDF composite membrane biofouling.	[121]
**MFC-MBR**	PVDF hollow fiber membrane of the MFC.	Backwashable conductive carbon nanotube (CNT) membrane deposited in PVDF membrane.	TMP rise to 30 kPa extended by 8 days. Reduction in phenol byproducts, cholesterol margarate, and carboxylic acid.	[126]
**Crossflow membrane filtration system**	Polyethersulfone (PES) membranes were CNT-coated with polyvinyl alcohol (PVA) crosslinked by succinic acid or glutaraldehyde.	Enhancing ECM stability in separation applications by examining antifouling properties of CNT/PVA-based ECMs with two crosslinking agents.	ECMs exhibited 21% flux reduction vs. 69% in PES membranes and 91% bacterial retention due to CNT porous structure.	[127]
**Filtration system**	(CNT)-PVDF conductive membrane	CNT-PVDF conductive membrane was tested in multi-cycle lab-scale filtration–backwashing experiments, with a DC voltage of −1.5 V applied across the membrane.	Significantly reduced irreversible biofouling, decreasing flux decline from 68% to 38% for live bacteria, effectively inhibited bacterial adhesion and enhanced backwashing, achieving up to 93% flux recovery.	[128]
**MFC-AnMBR**	PPy/AQDS/PTFE	Conductive anode membranes are used to treat Na^+^- and Mg^2+^-containing wastewater.	Reduction in protein and polysaccharide content by up to 44% and 58% for Na^+^ and up to 40% and 38% for Mg^2+^, while extending operational times for up to 43 days, with mitigation linked to current for Na^+^ and voltage for Mg^2+^.	[129]

Electro-assisted systems offer a promising, chemical-free approach to biofouling mitigation; however, their long-term success depends on managing operational risks such as electrode corrosion, by-product formation, and pH drift. Proactive design and operational protocols are essential to ensure durability and performance. The main operational risks are presented in Table 5.

## 4. Future Directions

While significant progress has been made in developing membrane modifications, nanocomposite membranes, and hybrid antifouling strategies, further research is essential to enhance long-term biofouling resistance and improve the scalability of these solutions. The following key areas should be prioritized:

- Material Innovation and Surface Engineering: The development of highly hydrophilic, conductive, and antimicrobial membranes is crucial for improving biofouling resistance. A promising approach is the incorporation of nanocomposite materials, such as functionalized graphene derivatives, metal–organic frameworks (MOFs), carbon nanotubes, and bio-inspired coatings, into the polymer matrix. Ceramic membranes have demonstrated superior chemical and mechanical stability, making them promising alternatives for long-term biofouling mitigation. Moreover, electro-assisted ceramic membranes can further enhance antifouling performance.
- Advanced Monitoring and Real-Time Diagnostics: Future studies should move beyond post hoc characterization and develop methods for the real-time, quantitative assessment of membrane health. A key direction is the integration of real-time diagnostic tools, such as polarization loss decomposition, to dynamically estimate the membrane’s health state. This approach can quantitatively separate and track the contribution of biofouling to membrane resistance, enabling proactive fouling management rather than reactive cleaning.
- System Operational Strategies and Thermal Management: Optimizing operational parameters is a vital complement to material-based solutions. Temperature control is a critical operational variable, as optimized thermal management can suppress microbial metabolic rates and EPS production, thereby delaying biofilm growth. Future research should explore thermal management strategies as a systematic approach to complement material-based antifouling approaches.
- Sustainability and Scalable Fabrication: Future research should focus on the development of low-cost, eco-friendly membrane materials and fabrication processes to ensure the long-term viability of BEC systems. This includes investigating green synthesis techniques, biodegradable membrane materials, and energy-efficient fabrication methods. Since even with efforts to develop anti-biofouling membranes that mitigate biofouling growth, membrane performance will still deteriorate over time, eventually requiring the replacement of fouled modules.
- Large-Scale Applications: Membrane modifications that demonstrated excellent performance in lab-scale and pilot-scale studies are essential to be tested under real-world, large-scale conditions to evaluate their long-term efficiency, durability, and practicality.
- Hybrid Systems: One should explore the integration of BECs with other technologies, such as forward osmosis [133], anaerobic membrane bioreactors, and electrochemical systems, to enhance biofouling resistance and overall system efficiency.
- Long-Term Studies: One should conduct extended operational studies to evaluate the durability and antifouling performance of membranes under real-world conditions.

## 5. Conclusions

This review has systematically synthesized and analyzed the most recent advancements (2019–2025) in membrane biofouling mitigation for Bioelectrochemical Cells (BECs). Moving beyond previous reviews, this work establishes its unique contribution through three key innovations that provide a forward-looking perspective on the field.

First, it provides the first systematic comparison and analysis of electro-assisted antifouling technology across BEC systems. The review consolidates evidence demonstrating that the application of low-intensity DC electric fields or the use of self-generated current in conductive membranes is a potent, chemical-free strategy. This approach mitigates biofouling through mechanisms like electrophoresis, electrostatic repulsion, and in situ electrochemical cleaning, significantly reducing extracellular polymeric substances (EPS) and extending operational cycles.

Second, this review introduces a critical economic and sustainability analysis of next-generation membranes, with a specific focus on ceramic and recycled material alternatives. While materials like functionalized graphene oxide and silver nanoparticles enhance performance, their scalability is often limited by cost. This work highlights those ceramic membranes, particularly when doped with elements like Cobalt (5CoBCGO), and low-cost composites using recycled materials (like polypropylene non-woven fabric or cigarette filter waste) offer a compelling balance of superior biofouling resistance, mechanical stability, and cost-effectiveness, positioning them as viable candidates for large-scale BEC deployment.

Third, the analysis offers an updated and consolidated evaluation of advanced material modifications, charting the clear evolution from simple hydrophilicity enhancement to multi-functional nanocomposites. The most effective strategies, such as incorporating antimicrobial nanoparticles (like Ag, CuO) with hydrophilic materials (like GO, zeolites) or designing smart coatings with quaternary ammonium compounds, successfully create synergistic effects that simultaneously repel and inactivate microorganisms, leading to a more robust and durable antifouling performance.

In conclusion, the future of membrane biofouling control lies in the intelligent integration of these avenues: developing smart, multi-functional materials, leveraging electrokinetic phenomena for in situ cleaning, and rigorously prioritizing cost-effective and sustainable membrane solutions. By systematically mapping these recent advancements, this review provides a critical foundation for guiding future research and development towards more efficient, durable, and commercially viable BEC systems.

## Figures and Tables

**Table 1:** The key properties of Nafion and the significance of these properties.

Property	Structural Origin	Significance in BEC Applications	Refs.
High Proton Conductivity	Nanoscale hydrophilic domains form interconnected ion-conducting pathways upon hydration.	Enables efficient transport of protons (H^+^) across the membrane, which is essential for cell performance.	[23,24]
Excellent Chemical Stability	Highly inert PTFE backbone resists chemical attack.	Provides long-term durability in the harsh electrochemical environments.	[25]
Strong Acidity	Sulfonic acid groups stabilized in perfluorinated matrix	Enhances proton dissociation and conduction.	[26]
Mechanical Strength	PTFE backbone and phase-separated morphology	Maintains membrane integrity under stress	[25]
Selectivity	Phase-separated structure limits crossover of gases and ions	Reduces fuel/oxidant crossover, improving efficiency	[27]

**Table 2:** Comparative performance of Nafion and alternative CEMs in MFCs [62,63].

Membrane	Cost (€ m^−2^)	Ion Exchange Capacity (IEC) (meq g^−1^)	Selectivity	Durability (Test Duration/Conditions)
Nafion 117	400	0.95–1.01	-	6 months at 30 °C, pH 7; 37% power loss due to fouling
CMI-7000	170	1.6	>0.97%	120 days; roughness increased, indicating fouling
Zirfon^®^	45	-	-	120 days; minimal fouling and stable conductivity
FKB	320	0.9–1.0	>0.98%	120 days; slow fouling progression
FKE	195	>1	>0.98%	120 days; stable mechanical and chemical resistance

**Table 3:** Biofouling mitigation studies with membrane modification.

Type of the Cell	Membrane Type	Maximum Power Density (mW/m^2^)	H_2_ Production	Main Idea	Biofouling Capabilities	Ref.
**Tabular MFC**	SPSEBS +6% SPOSS	126		Studying the biofilm formation on SPOSS-incorporated SPSEBS nanocomposites at different weight percentages of nano fillers (2–8%)	Biofouling results explain that the addition of nanofiller reduces the biofilm formation and increases the proton conductivity.	[13]
**Two-Chamber MEC**	Nafion-117, CMI-7000, Zirfon UTP 500, FKE, and FKB	Constant power density of around 1000	-	Studying mechanical, chemical, and electrochemical properties of five commercially available CEMs developed over four months in MEC.	Over four months, fouling increased membrane roughness, with CMI and Nafion rising from 7 nm to 26 nm and 23 nm, respectively, while Zirfon and FKB/FKE showed slower increases.	[64]
**Direct Methanol Fuel Cell (DMFC)**	Nafion^®^/S-ZrO_2_ (NH_3_SO_4_) and Nafion^®^/S-ZrO_2_ membranes	Maximum power density Nafion^®^/S-ZrO_2_ (NH_3_SO_4_): 1.83 × 10^6^ and Nafion^®^/S-ZrO_2_: 1.58 × 10^6^		Modifying Nafion^®^ membranes for fuel cells by incorporating sulfated zirconium compared to commercial Nafion^®^ 117.	he Nafion^®^/S-ZrO_2_ (NH_3_SO_4_) membranes showed improved proton conductivity (7.891 S/cm), better hydrophilicity (contact angles reduced to 68°).	[66]
**Two-Chamber MEC**	CMI7000		0.2 LH_2_/L_reactor_·day with a purity always higher than 98%	Investigating the durability of cation exchange membrane (CEM) in MECs operated in fed-batch mode for a period of 78 days and used for hydrogen production and ammonia recovery from pig slurry.	The membrane’s Ion Exchange Capacity (IEC) dropped by up to 22% at the inlet and outlet, while biofouling caused increased surface roughness, especially in samples M1, M2, and M6.	[67]
**Single-Chamber MFC**	(fumasep^®^ FFFA-3-30, FUMATECH BWT GmbH, Bietigheim-Bissingen, Germany)	200		A membrane-electrode assembly was tested using an antibacterial membrane affixed to a gas diffusion electrode.	A thin biofilm developed on the electrode surface but was not cross-linked to the membrane, allowing for easier removal.	[68]
**Single-Chamber AC-MFC**	PP-373 and PP-468	81	-	Developing a ceramic membrane using recycled polypropylene on ceramic materials.	PP-373 maintained the lowest contact angle over time and neglectable weight gain after operation.	[71]
**MFC**	BCGO, 3LiBCGO and 5CoBCGO	-	-	To dope BCGO (ceramic membrane) with two different materials (Co and Li).	Yeast cells are prevented from crossing the membranes with ions. 5CoBCGO had better biofouling mitigation capabilities with the thinnest thickness of biofilm.	[77]
**Two-Chamber MFC**	Bio-cellulose-[BMIM][Cl]	~173		Comparing ionogel membrane efficiency and biofilm behavior against a Nafion membrane, both equipped in an acetate-fed MFC.	The ionogel membrane boosted microbial growth but had higher biofouling and resistance, lowering MFC performance compared to Nafion.	[83]
**Two-Chamber MFC**	(GO-SPEEK) and (AgGO-GO-SPEEK).	1049 for AgGO-GO-SPEEK	-	GO enhances the mechanical structure of the polymeric membrane as well as the proton conductivity. Ag-Go is an antibacterial material.	AgGO-GO-SPEEK exhibits the lowest internal resistance with 23.83%. GO hydrophilicity and Ag antibacterial activity.	[87]
**MFC**	SPEEK/SSA + 1.5 wt.% CuO@rGO as membrane filler	186 ± 5	-	The SPEEK/SSA hydrophilicity and CuO@rGO antibacterial properties improved the biofouling mitigation.	The membrane had a biofilm thickness of 77 ± 0.6 micrometers and a contact angle of 34.6°.	[88]
**Two-Chamber MFC**	CuGNSs (2.0)/SPES	84.1	-	The use of CuGNSs as modifier agents in PEM, which were biosynthesized from plants.	The membrane obtained a contact angle of 59.7°, and a high negative zeta potential was obtained for PH higher than 5 (almost −70 mV for pH = 7.5)	[89]
**Two-Chamber MFC**	Zeolite 4A to the PVC matrix (PVC-Zeolite membranes)	250 ± 5 at 340 mA/m^2^ current density.	-	The use of zeolite 4A hydrophilicity with the hydrophobicity of PVC matrix	Biofilm attachment was minimized due to antibacterial properties and high hydrophilicity of the composite membrane.	[86]
**Two-Chamber MEC**	AgNP-PDA/SPAES/PIN	-	Hydrogen production maintained by a reduction of 31.88%	SPAES/PIN-PEM modified by surface functionalization with AgNP and PDA, multiple coating types and orders were investigated.	The biofilm formation was reduced by 80.74% relative to the pristine membrane. The contact angle of PDA-Ag 0.036 was 37.41°.	[97]
**MFC**	SPEEK + 7.5 wt.% Ag	156 ± 0.5	-	SPEEK with (2.5, 5, 7.5 or 10 wt.%) Ag additives was investigated.	The presence of biofilm formation was reduced. The addition of silver increased power density by mitigating biofouling over time.	[98]
**MFC**	SPEEK/CeO_2_-AtiO_2_ (2%)	Maximum power density: 1.17 × 10^6^ at 100% Relative Humidity (RH)	-	The incorporation of (CeO_2_-ATiO_2_) as a bifunctional nanofiller i00n SPEEK to reduce cost and enhance performance.	Durability of the composite membrane was enhanced, OCV decay of 0.925 mV/h at T = 60 °C and RH = 30%.	[105]
**Two-Chamber H-type MFC**	Nafion 117	Maximum power density of rice straw: 119.35 mW/m^2^ potato peels: 152.55 mW/m^2^	-	The biofouling effect was investigated using SEM.	A reduction in power generation was observed after biofouling by 37%. Biofouling was highly detectable.	[63]
**Osmotic Microbial Fuel Cell (OsMFC)**	AgNP modified thin film composite (TFC) polyamide FO membrane	3.67 W/m^2^	-	Advanced hydrophilicity, more negative zeta potential and better antibacterial properties led to biofouling mitigation.	A decrease of 25% in the number of *E. coli* bacteria colonies was observed	[10]
**Two-Chamber MFC**	PSEBS SU22 PSEBS-based, sulfomethylated, CEM membrane	149 mW/m^2^ at 595 mA/m^−2^	-	Biofouling was mechanically removed and followed by chemical conditioning.	Minor differences could be observed between the fouling layers of Nafion 115 and PSEBS SU22	[85]
**Two-Chamber MEC**	PDA-PEI-Ag@PVDF		2.65 ± 0.02 m^3^/m^3^/d. An overall hydrogen recovery of 86–94%.	Improving hydrogen production in MEC by enhancing PVDF membranes with a PDA, PEI, and AgNP coating.	An improved hydrophilicity with a contact angle of 8.0 ± 1°. The membrane exhibited high antibacterial activity due to the formation of AgNPs in comparison to a pristine membrane.	[106]
**Two-Chamber MFC**	PSEBS DABCO	Current Density: AEM: 400 mA/m^2^ at Acetate concentration = 5 mM. PEM: 285 mA/m^2^ at Acetate concentration = 5 mM.	-	Comparing the performance of PEM (Nafion) and AEM (PSEBS-DABCO), combining the physical properties of PSEBS and the chemical properties	AEM showed a lower internal resistance of 145 Ω, which was lower than PEM by 194 Ω. PEM thickness increased by 16%, whereas AEM thickness changes were negligible.	[107]
**Two-Chamber MFC**	SPEEK/SSA + 5% SGO	203 mW/m^2^		Investigates the development of SGO functionalized PEM for MFCs to enhance power generation and mitigate biofouling	31.52% biofilm inhibition, enhanced hydrophilicity (33.5° water contact angle)	[108]
**Filtration cell/system**	PDA/p(DM)-3-PVDF	-	-	Ultrafiltration PVDF membranes were coated with zwitterionic (MPC, CBMA) and cationic (DMC) copolymers for dual antifouling and antibacterial performance.	(PDA/p(DCD)-1-PVDF) achieved BSA rejection of more than 97%, flux recovery 84.78%, FDR = 9.57%, and 100% antibacterial efficiency.	[101]
**-**	W-CBF 2	-	-	Recycled cigarette filter waste modified with tannic acid and FeCl_3_ coatings was used to develop a sustainable antifouling ultrafiltration membrane	Flux recovery ratios of 85% and 76%, with reduced irreversible fouling and increased reversible fouling.	[72]
**Anaerobic/Oxic MBR**	M-CNTs/PDA	-	-	CNTs/PDA- membrane was fabricated and systematically evaluated its performance in a continuous-flow A/O-MBR.	M-CNTs/PDA achieved high nitrogen removal efficiency (~85%), maintained a 30–40% higher permeability, and demonstrated stronger antifouling performance with over 70% flux recovery.	[109]
**Four-Chamber Test Cell**	ZIF-8/CS			Chitosan (CS), a cheap, biodegradable biopolymer, was combined with ZIF-8 to create a composite material.	High ionic conductivity of 0.099 S/cm, competing with Nafion-117 (0.13 S/cm), and only a 25.88% increase in surface roughness compared to Nafion’s 179.48%	[92]
**Four-Chamber Test Cell**	Chitosan-based membranes doped with Er/Al/ZnO			Fabricated and tested photocatalyst-enhanced chitosan membranes with Er/Al/ZnO improve ionic conductivity and resistance to fouling and biofouling	Surface resistance dropped f to 6.84 Ω·cm^2^ (MQ), 5.15 Ω·cm^2^ (MQ1), and 5.06 Ω·cm^2^ (MQ2), biofouling resistance improved from 13.09 v to 8.06 Ω·cm^2^, and Nafion fouled 10.21 more	[93]
**MFC**	Sulfonation + PP13-TFSI on PVDF-co-HFP	325 mW/m^2^		PVDF-co-HFP was modified via sulfonation and ionic liquid (PP13-TFSI) incorporation	Achieved 22.8% water uptake, 1.25 meq/g IEC, 10.83 mS/cm proton conductivity, 54.1° contact angle, and minimal performance loss over 5 cycles and a cost saving of $188–1388 per m^2^	[73]
**MEC**	PDMS-modified Cellophane			(PDMS)-modified Cellophane separator was tested for its low cost, effectively maintains a stable pH environment, and exhibits manageable energy loss	Costs only 10% of a commercial membrane, doubles the lifespan of plain Cellophane from 1 to 2 months, and effectively prevents pH imbalance like an AEM	[75]
**Membrane Cathode Assembly (MCA) single Chamber MFC**	PVA–NH_4_I–EMMIB			Investigated the development of cheap ionic liquid-doped PVA anion exchange membrane.	High ionic conductivity of 2.4 × 10^−2^ S/cm at the optimal loading, which is a 26-fold increase over the plain PVA membrane, and reducing cell biomass by over 60% compared to the control.	[84]
**MFC**	SPEEK/SSA + SPMA	196.6 mW/m^2^		Studied novel nanocomposite membrane (SPEEK/SSA with phosphomolybdic acid-functionalized silica.	Achieved proton conductivity of 3.75 × 10^−2^ S cm and a significant 67.52% biofilm inhibition.	[74]
	GO-PSF			Created a thin, top layer of GO mixed with polysulfone (PSF) on a standard PSF support.	Improved hydrophilicity (contact angle 47.29° vs. 75.9°), demonstrated superior antifouling performance (83% vs. 52% flux recovery), and offered excellent antibacterial properties.	[94]

**Table 5:** Main operational risks.

Risk Factor	Description and Impact	Mitigation Strategies	Ref.
Electrode Corrosion	Electrochemical oxidation and reverse currents degrade electrodes, reducing lifespan.	Advanced electrode materials (like MXene/carbon nanofiber, corrosion-resistant coatings), periodic polarity reversal, and real-time monitoring.	[130]
By-Product Formation	Generation of free chlorine, mineral scales, or other undesirable compounds.	Physical barrier layers, optimized voltage/current control, selective catalysts, and electrolyte additives.	[131]
pH Drift	Water electrolysis causes local pH changes, affecting microbial inactivation and scaling.	Buffered electrolytes, careful voltage/current control, and system design to manage local pH.	[132]

## Data Availability

The original contributions presented in this study are included in this article. Further inquiries can be directed to the corresponding author.

## References

[1] Gross R., Leach M., Bauen A. (2003). Progress in renewable energy. Environ. Int..

[2] Dincer I. (2000). Renewable energy and sustainable development: A crucial review. Renew. Sustain. Energy Rev..

[3] Stoppato A., Benato A. (2017). The Importance of Energy Storage. Energy Storage.

[4] Alinejad Z., Parham N., Tawalbeh M., Al-Othman A., Almomani F. (2025). Progress in green hydrogen production and innovative materials for fuel cells: A pathway towards sustainable energy solutions. Int. J. Hydrogen Energy.

[5] Mei J., Meng X., Tang X., Li H., Hasanien H., Alharbi M., Dong Z., Shen J., Sun C., Fan F. (2024). An Accurate Parameter Estimation Method of the Voltage Model for Proton Exchange Membrane Fuel Cells. Energies.

[6] Torrisi S., Anastasi E., Longhitano S., Longo I.C., Zerbo A., Borzi G. Circular Economy and the Benefits of Biomass as a Renewable Energy Source. Proceedings of the 22th International Trade Fair of Material & Energy Recovery and Sustainable Development, ECOMONDO.

[7] Sonawane J.M., Ezugwu C.I., Ghosh P.C. (2020). Microbial Fuel Cell-Based Biological Oxygen Demand Sensors for Monitoring Wastewater: State-of-the-Art and Practical Applications. ACS Sens..

[8] Bennetto H.P. (1990). Electricity generation by microorganisms. Biotechnol. Educ..

[9] Rabaey K., Rozendal R.A. (2010). Microbial electrosynthesis—Revisiting the electrical route for microbial production. Nat. Rev. Microbiol..

[10] Lu Y., Jia J., Miao H., Ruan W., Wang X. (2020). Performance Improvement and Biofouling Mitigation in Osmotic Microbial Fuel Cells via In Situ Formation of Silver Nanoparticles on Forward Osmosis Membrane. Membranes.

[11] Tawalbeh M., Al-Othman A., Singh K., Douba I., Kabakebji D., Alkasrawi M. (2020). Microbial desalination cells for water purification and power generation: A critical review. Energy.

[12] Ji M., Wang J. (2021). Review and comparison of various hydrogen production methods based on costs and life cycle impact assessment indicators. Int. J. Hydrogen Energy.

[13] Sugumar M., Dharmalingam S. (2020). Statistical optimization of process parameters in microbial fuel cell for enhanced power production using Sulphonated Polyhedral Oligomeric Silsesquioxane dispersed Sulphonated Polystyrene Ethylene Butylene Polystyrene nanocomposite membranes. J. Power Sources.

[14] Mohan S.V., Sravan J.S., Butti S.K., Krishna K.V., Modestra J.A., Velvizhi G., Kumar A.N., Varjani S., Pandey A. (2019). Microbial Electrochemical Technology. Microbial Electrochemical Technology.

[15] Al-Murisi M., Al-Muqbel D., Al-Othman A., Tawalbeh M. (2022). Integrated microbial desalination cell and microbial electrolysis cell for wastewater treatment, bioelectricity generation, and biofuel production: Success, experience, challenges, and future prospects. Integrated Environmental Technologies for Wastewater Treatment and Sustainable Development.

[16] Benemann J. (1996). Hydrogen biotechnology: Progress and prospects. Nat. Biotechnol..

[17] Ki D., Popat S.C., Torres C.I. (2016). Reduced overpotentials in microbial electrolysis cells through improved design, operation, and electrochemical characterization. Chem. Eng. J..

[18] Jeremiasse A.W., Hamelers H.V.M., Buisman C.J.N. (2010). Microbial electrolysis cell with a microbial biocathode. Bioelectrochemistry.

[19] Rasten E., Hagen G., Tunold R. (2003). Electrocatalysis in water electrolysis with solid polymer electrolyte. Electrochim. Acta.

[20] Sharma A., Hussain Mehdi S.E., Pandit S., Eun-Oh S., Natarajan V. (2024). Factors affecting hydrogen production in microbial electrolysis cell (MEC): A review. Int. J. Hydrogen Energy.

[21] Zhang Y., Angelidaki I. (2014). Microbial electrolysis cells turning to be versatile technology: Recent advances and future challenges. Water Res..

[22] Gautam R., Nayak J.K., Ress N.V., Steinberger-Wilckens R., Ghosh U.K. (2023). Bio-hydrogen production through microbial electrolysis cell: Structural components and influencing factors. Chem. Eng. J..

[23] Xu G., Dong X., Xue B., Huang J., Wu J., Cai W. (2023). Recent Approaches to Achieve High Temperature Operation of Nafion Membranes. Energies.

[24] Safronova E.Y., Voropaeva D.Y., Lysova A.A., Korchagin O.V., Bogdanovskaya V.A., Yaroslavtsev A.B. (2022). On the Properties of Nafion Membranes Recast from Dispersion in N-Methyl-2-Pyrrolidone. Polymers.

[25] Shi S., Weber A.Z., Kusoglu A. (2016). Structure/property relationship of Nafion XL composite membranes. J. Memb. Sci..

[26] Lufrano E., Simari C., Di Vona M.L., Nicotera I., Narducci R. (2021). How the Morphology of Nafion-Based Membranes Affects Proton Transport. Polymers.

[27] Min K., Al Munsur A.Z., Paek S.Y., Jeon S., Lee S.Y., Kim T.-H. (2023). Development of High-Performance Polymer Electrolyte Membranes through the Application of Quantum Dot Coatings to Nafion Membranes. ACS Appl. Mater. Interfaces.

[28] Sharma A., Đelević L., Herkendell K. (2024). Next-Generation Proton-Exchange Membranes in Microbial Fuel Cells: Overcoming Nafion’s Limitations. Energy Technol..

[29] Miskan M., Ismail M., Ghasemi M., Md Jahim J., Nordin D., Abu Bakar M.H. (2016). Characterization of membrane biofouling and its effect on the performance of microbial fuel cell. Int. J. Hydrogen Energy.

[30] Jadhav D.A., Pandit S., Sonawane J.M., Gupta P.K., Prasad R., Chendake A.D. (2021). Effect of membrane biofouling on the performance of microbial electrochemical cells and mitigation strategies. Bioresour. Technol. Rep..

[31] Costerton J.W., Lewandowski Z., Debeer D., Caldwell D., Korber D., James2 G. (1994). MINIREVIEW Biofilms, the Customized Microniche. J. Bateriol..

[32] Lin H., Zhang M., Wang F., Meng F., Liao B.-Q., Hong H., Chen J., Gao W. (2014). A critical review of extracellular polymeric substances (EPSs) in membrane bioreactors: Characteristics, roles in membrane fouling and control strategies. J. Memb. Sci..

[33] Lin H., Wang F., Ding L., Hong H., Chen J., Lu X. (2011). Enhanced performance of a submerged membrane bioreactor with powdered activated carbon addition for municipal secondary effluent treatment. J. Hazard. Mater..

[34] Satyawali Y., Balakrishnan M. (2009). Effect of PAC addition on sludge properties in an MBR treating high strength wastewater. Water Res..

[35] Jamal Khan S., Visvanathan C., Jegatheesan V. (2012). Effect of powdered activated carbon (PAC) and cationic polymer on biofouling mitigation in hybrid MBRs. Bioresour. Technol..

[36] Sheng G.P., Yu H.Q., Yue Z. (2006). Factors influencing the production of extracellular polymeric substances by Rhodopseudomonas acidophila. Int. Biodeterior. Biodegrad..

[37] Ye F., Peng G., Li Y. (2011). Influences of influent carbon source on extracellular polymeric substances (EPS) and physicochemical properties of activated sludge. Chemosphere.

[38] Fuqua W.C., Winans S.C., Greenberg E.P. (1994). Quorum sensing in bacteria: The LuxR-LuxI family of cell density-responsive transcriptional regulators. J. Bacteriol..

[39] Siddiqui M.F., Rzechowicz M., Harvey W., Zularisam A.W., Anthony G.F. (2015). Quorum sensing based membrane biofouling control for water treatment: A review. J. Water Process Eng..

[40] Muras A., Parga A., Mayer C., Otero A. (2021). Use of Quorum Sensing Inhibition Strategies to Control Microfouling. Mar. Drugs.

[41] Ham S.-Y., Kim H.-S., Cha E., Park J.-H., Park H.-D. (2018). Mitigation of membrane biofouling by a quorum quenching bacterium for membrane bioreactors. Bioresour. Technol..

[42] Bouayed N., Dietrich N., Lafforgue C., Lee C.-H., Guigui C. (2016). Process-Oriented Review of Bacterial Quorum Quenching for Membrane Biofouling Mitigation in Membrane Bioreactors (MBRs). Membranes.

[43] Xu J., Sheng G.-P., Luo H.-W., Li W.-W., Wang L.-F., Yu H.-Q. (2012). Fouling of proton exchange membrane (PEM) deteriorates the performance of microbial fuel cell. Water Res..

[44] Ghasemi M., Wan Daud W.R., Ismail M., Rahimnejad M., Ismail A.F., Leong J.X., Miskan M., Ben Liew K. (2013). Effect of pre-treatment and biofouling of proton exchange membrane on microbial fuel cell performance. Int. J. Hydrogen Energy.

[45] Xia L., Vemuri B., Saptoka S., Shrestha N., Chilkoor G., Kilduff J., Gadhamshetty V. (2019). Antifouling Membranes for Bioelectrochemistry Applications. Microbial Electrochemical Technology.

[46] Tawalbeh M., Qalyoubi L., Al-Othman A., Qasim M., Shirazi M. (2023). Insights on the development of enhanced antifouling reverse osmosis membranes: Industrial applications and challenges. Desalination.

[47] Belfort G., Davis R.H., Zydney A.L. (1994). The behavior of suspensions and macromolecular solutions in crossflow microfiltration. J. Memb. Sci..

[48] Zuo K., Chen M., Liu F., Xiao K., Zuo J., Cao X., Zhang X., Liang P., Huang X. (2018). Coupling microfiltration membrane with biocathode microbial desalination cell enhances advanced purification and long-term stability for treatment of domestic wastewater. J. Memb. Sci..

[49] Vermaas D.A., Kunteng D., Veerman J., Saakes M., Nijmeijer K. (2014). Periodic Feedwater Reversal and Air Sparging As Antifouling Strategies in Reverse Electrodialysis. Environ. Sci. Technol..

[50] Ostuni E., Chapman R.G., Holmlin R.E., Takayama S., Whitesides G.M. (2001). A Survey of Structure−Property Relationships of Surfaces that Resist the Adsorption of Protein. Langmuir.

[51] Zhou M., Liu H., Kilduff J.E., Langer R., Anderson D.G., Belfort G. (2009). High-throughput membrane surface modification to control NOM fouling. Environ. Sci. Technol..

[52] Tawalbeh M., Aljaghoub H., Qasim M., Al-Othman A. (2023). Surface modification techniques of membranes to improve their antifouling characteristics: Recent advancements and developments. Front. Chem. Sci. Eng..

[53] Kang G.D., Cao Y.M. (2012). Development of antifouling reverse osmosis membranes for water treatment: A review. Water Res..

[54] Zhao C., Xue J., Ran F., Sun S. (2013). Modification of polyethersulfone membranes—A review of methods. Prog. Mater. Sci..

[55] Villalobos García J., Dow N., Milne N., Zhang J., Naidoo L., Gray S., Duke M. (2018). Membrane Distillation Trial on Textile Wastewater Containing Surfactants Using Hydrophobic and Hydrophilic-Coated Polytetrafluoroethylene (PTFE) Membranes. Membranes.

[56] Pasternak G., De Rosset A., Tyszkiewicz N., Widera B., Greenman J., Ieropoulos I. (2022). Prevention and removal of membrane and separator biofouling in bioelectrochemical systems: A comprehensive review. iScience.

[57] Nasruddin N.I.S.M., Abu Bakar M.H. (2021). Mitigating membrane biofouling in biofuel cell system-A review. Open Chem..

[58] Koók L., Bakonyi P., Harnisch F., Kretzschmar J., Chae K.-J., Zhen G., Kumar G., Rózsenberszki T., Tóth G., Nemestóthy N. (2019). Biofouling of membranes in microbial electrochemical technologies: Causes, characterization methods and mitigation strategies. Bioresour. Technol..

[59] Desmond P., Huisman K.T., Sanawar H., Farhat N.M., Traber J., Fridjonsson E.O., Johns M.L., Flemming H.-C., Picioreanu C., Vrouwenvelder J.S. (2022). Controlling the hydraulic resistance of membrane biofilms by engineering biofilm physical structure. Water Res..

[60] Roy H., Rahman T.U., Tasnim N., Arju J., Rafid M.M., Islam M.R., Pervez M.N., Cai Y., Naddeo V., Islam M.S. (2023). Microbial Fuel Cell Construction Features and Application for Sustainable Wastewater Treatment. Membranes.

[61] Fan L., Shi J., Xi Y. (2020). PVDF-Modified Nafion Membrane for Improved Performance of MFC. Membranes.

[62] Noori M.T., Ghangrekar M.M., Mukherjee C.K., Min B. (2019). Biofouling effects on the performance of microbial fuel cells and recent advances in biotechnological and chemical strategies for mitigation. Biotechnol. Adv..

[63] Flimban S.G.A., Hassan S.H.A., Rahman M.M., Oh S.E. (2020). The effect of Nafion membrane fouling on the power generation of a microbial fuel cell. Int. J. Hydrogen Energy.

[64] San-Martín M.I., Carmona F.J., Alonso R.M., Prádanos P., Morán A., Escapa A. (2019). Assessing the ageing process of cation exchange membranes in bioelectrochemical systems. Int. J. Hydrogen Energy.

[65] Inamuddin, Shakeel N., Imran Ahamed M., Kanchi S., Abbas Kashmery H. (2020). Green synthesis of ZnO nanoparticles decorated on polyindole functionalized-MCNTs and used as anode material for enzymatic biofuel cell applications. Sci. Rep..

[66] Sigwadi R., Mokrani T., Dhlamini M.S., Nonjola P., Msomi P.F. (2019). Nafion^®^/ sulfated zirconia oxide-nanocomposite membrane: The effects of ammonia sulfate on fuel permeability. J. Polym. Res..

[67] San-Martín M.I., Sotres A., Alonso R.M., Díaz-Marcos J., Morán A., Escapa A. (2019). Assessing anodic microbial populations and membrane ageing in a pilot microbial electrolysis cell. Int. J. Hydrogen Energy.

[68] Haupt D.R., Landwehr L., Schumann R., Hahn L., Issa M., Coskun C., Kunz U., Sievers M. (2022). A New Reactor Concept for Single-Chamber Microbial Fuel Cells and Possible Anti-Fouling Strategies for Long-Term Operation. Microorganisms.

[69] Wang C., Yang F., Meng F., Zhang H., Xue Y., Fu G. (2010). High flux and antifouling filtration membrane based on non-woven fabric with chitosan coating for membrane bioreactors. Bioresour. Technol..

[70] Zhao J., Shi Q., Luan S., Song L., Yang H., Shi H., Jin J., Li X., Yin J., Stagnaro P. (2011). Improved biocompatibility and antifouling property of polypropylene non-woven fabric membrane by surface grafting zwitterionic polymer. J. Memb. Sci..

[71] Pasternak G., Ormeno-Cano N., Rutkowski P. (2021). Recycled waste polypropylene composite ceramic membranes for extended lifetime of microbial fuel cells. Chem. Eng. J..

[72] Luthfiana A., Mulyati S., Arahman N., Bilad M.R., Aulia M.P. (2025). Cigarette butt filter as membrane material with tannic acid and FeCl3 additives for improve antifouling properties. Case Stud. Chem. Environ. Eng..

[73] Sun B., Pan X., Tian Y., Bi W., Feng M., Liu F., Hou Q. (2025). Enhancement of electrochemical performance in PVDF-co-HFP cation exchange membrane with modifications by doping PP13-TFSI ionic liquid and sulfonation. Environ. Technol. Innov..

[74] Solomon J., Dharmalingam S. (2024). Phosphomolybdic acid functionalized nano silica for enhanced anti-biofouling effect in polymer electrolyte membranes for microbial fuel cell application. Process Saf. Environ. Prot..

[75] Colantoni S., Pillot G., Cvoro S., Kerzenmacher S., Santiago Ó. (2025). Evaluation of separators for potential use in microbial electrolysis cells under anaerobic digester conditions. J. Memb. Sci..

[76] Omar N.M.A., Othman M.H.D., Tai Z.S., Kurniawan T.A., Puteh M.H., Jaafar J., Rahman M.A., Ismail A.F., Rajamohan N., Abdullah H. (2024). Recent strategies for enhancing the performance and lifespan of low-cost ceramic membranes in water filtration and treatment processes: A review. J. Water Process Eng..

[77] Frattini D., Accardo G., Kwon Y. (2020). Perovskite ceramic membrane separator with improved biofouling resistance for yeast-based microbial fuel cells. J. Memb. Sci..

[78] Koók L., Nemestóthy N., Bakonyi P., Göllei A., Rózsenberszki T., Takács P., Salekovics A., Kumar G., Bélafi-Bakó K. (2017). On the efficiency of dual-chamber biocatalytic electrochemical cells applying membrane separators prepared with imidazolium-type ionic liquids containing [NTf2]− and [PF6]− anions. Chem. Eng. J..

[79] Hernández-Fernández F.J., Pérez de los Ríos A., Mateo-Ramírez F., Godínez C., Lozano-Blanco L.J., Moreno J.I., Tomás-Alonso F. (2015). New application of supported ionic liquids membranes as proton exchange membranes in microbial fuel cell for waste water treatment. Chem. Eng. J..

[80] Alashkar A., Al-Othman A., Tawalbeh M., Qasim M. (2022). A Critical Review on the Use of Ionic Liquids in Proton Exchange Membrane Fuel Cells. Membranes.

[81] Grzybek P., Dudek G., van der Bruggen B. (2024). Cellulose-based films and membranes: A comprehensive review on preparation and applications. Chem. Eng. J..

[82] He S., Kamio E., Zhang J., Matsuoka A., Nakagawa K., Yoshioka T., Matsuyama H. (2023). Development of an ion gel membrane containing a CO_2_-philic ionic liquid in interpenetrating semi-crystalline and crosslinkable polymer networks. J. Memb. Sci..

[83] Szakács S., Martínez E.O., Koók L., Santos G.M., Alarcon J.T., Jeison D., Pientka Z., Nemestóthy N., Bélafi-Bakó K., Bakonyi P. (2024). Biofouling-focused assessment of a novel, cellulose-based ionogel membrane applied in a microbial fuel cell. Bioresour. Technol. Rep..

[84] Tomar R., Chandra S., Pandit S., Shahid M., Sharma K., Raj S., Geetha S.J., Joshi S.J. (2025). Novel ionic liquid-infused PVA-based anion exchange membranes boosting bioelectricity yield from microbial fuel cells. Heliyon.

[85] Koók L., Žitka J., Szakács S., Rózsenberszki T., Otmar M., Nemestóthy N., Bélafi-Bakó K., Bakonyi P. (2021). Efficiency, operational stability and biofouling of novel sulfomethylated polystyrene-block-poly(ethylene-ran-butylene)-block-polystyrene cation exchange membrane in microbial fuel cells. Bioresour. Technol..

[86] Nagar H., Badhrachalam N., Rao V.V.B., Sridhar S. (2019). A novel microbial fuel cell incorporated with polyvinylchloride/4A zeolite composite membrane for kitchen wastewater reclamation and power generation. Mater. Chem. Phys..

[87] Ben Liew K., Leong J.X., Wan Daud W.R., Ahmad A., Hwang J.J., Wu W. (2020). Incorporation of silver graphene oxide and graphene oxide nanoparticles in sulfonated polyether ether ketone membrane for power generation in microbial fuel cell. J. Power Sources.

[88] Solomon J., Dharmalingam S. (2023). Modified polymer electrolyte membrane for microbial fuel cell: Performance analysis, investigation on anti-biofouling effect, and microbial community analysis on biofouled membrane. J. Power Sources.

[89] Shirvani B., Rahimi M., Zinadini S. (2023). High electricity generation and wastewater treatment enhancement using a microbial fuel cell equipped with conductive and anti-biofouling CuGNSs/SPES proton exchange membrane. Energy Convers. Manag..

[90] Asadollahi M., Bastani D., Musavi S.A. (2017). Enhancement of surface properties and performance of reverse osmosis membranes after surface modification: A review. Desalination.

[91] Hosseini Z., Kargari A. (2024). Fabrication and characterization of hydrophobic/hydrophilic dual-layer polyphenyl sulfone Janus membrane for application in direct contact membrane distillation. Desalination.

[92] Pupiales H., Soria R.B., Arboleda D., Cevallos C., Alcívar C., Francis L., Xu X., Luis P. (2025). ZIF-8/Chitosan Composite Hydrogel as a High-Performance Separator for Bioelectrochemical Systems. Membranes.

[93] Alvear Méndez S., Soria R.B., Arboleda D., Cevallos C., Alcívar C., Jimenez Y., Teran R., Pupiales H., Luis P. (2025). Influence of Er- and Al-Doped ZnO on Mixed-Matrix Membranes of Chitosan Derivatives in Bioelectrochemical Systems. Molecules.

[94] Xu X., Wang Q., Zhu X., Wu Q., Zheng T., Yuan H., Zhou Z. (2023). The preparation of anti-fouling dual-layer composite membrane with embedding graphene oxide. Asia-Pac. J. Chem. Eng..

[95] Ahmed S., Ahmad M., Swami B.L., Ikram S. (2016). A review on plants extract mediated synthesis of silver nanoparticles for antimicrobial applications: A green expertise. J. Adv. Res..

[96] Noori M.T., Tiwari B.R., Mukherjee C.K., Ghangrekar M.M. (2018). Enhancing the performance of microbial fuel cell using Ag Pt bimetallic alloy as cathode catalyst and anti-biofouling agent. Int. J. Hydrogen Energy.

[97] Park S.G., Rajesh P.P., Hwang M.H., Chu K.H., Cho S., Chae K.J. (2021). Long-term effects of anti-biofouling proton exchange membrane using silver nanoparticles and polydopamine on the performance of microbial electrolysis cells. Int. J. Hydrogen Energy.

[98] Kugarajah V., Dharmalingam S. (2021). Effect of silver incorporated sulphonated poly ether ether ketone membranes on microbial fuel cell performance and microbial community analysis. Chem. Eng. J..

[99] Ping M., Zhang X., Liu M., Wu Z., Wang Z. (2019). Surface modification of polyvinylidene fluoride membrane by atom-transfer radical-polymerization of quaternary ammonium compound for mitigating biofouling. J. Memb. Sci..

[100] Chen M., Zhang X., Wang Z., Wang L., Wu Z. (2017). QAC modified PVDF membranes: Antibiofouling performance, mechanisms, and effects on microbial communities in an MBR treating municipal wastewater. Water Res..

[101] Li T., Dong P., Zhang Q., Xiao H., Zheng H., Li L., Cao Z., Zhang M., Ma C., Xie G. (2025). Anti-adhesive and antimicrobial coatings on ultrafiltration membranes via dopamine-assisted co-deposition of zwitterionic-cationic copolymers. Colloids Surf. A Physicochem. Eng. Asp..

[102] Ng M.K., Mont M.A., Bonutti P.M. (2025). Clinical and Environmental Harms of Quaternary Ammonium Disinfectants and the Promise of Ultraviolet-C (UV-C) Alternatives: A Narrative Review. Cureus.

[103] EPA Drinking Water Regulations and Contaminants. Safe Drinking Water Act. https://www.epa.gov/sdwa/drinking-water-regulations-and-contaminants.

[104] Khanzada N.K., Al-Juboori R.A., Khatri M., Ahmed F.E., Ibrahim Y., Hilal N. (2024). Sustainability in Membrane Technology: Membrane Recycling and Fabrication Using Recycled Waste. Membranes.

[105] Ranganathan H., Vinothkannan M., Kim A.R., Subramanian V., Oh M.S., Yoo D.J. (2022). Simultaneous improvement of power density and durability of sulfonated poly(ether ether ketone) membrane by embedding CeO_2_-ATiO_2_: A comprehensive study in low humidity proton exchange membrane fuel cells. Int. J. Energy Res..

[106] Zhao N., Meng S., Li X., Liu H., Liang D. (2024). Enhancing proton transport in polyvinylidenedifluoride membranes and reducing biofouling for improved hydrogen production in microbial electrolysis cells. Bioresour. Technol..

[107] Koók L., Žitka J., Bakonyi P., Takács P., Pavlovec L., Otmar M., Kurdi R., Bélafi-Bakó K., Nemestóthy N. (2020). Electrochemical and microbiological insights into the use of 1,4-diazabicyclo[2.2.2]octane-functionalized anion exchange membrane in microbial fuel cell: A benchmarking study with Nafion. Sep. Purif. Technol..

[108] Solomon J., Ganesh N., Sundaram C.M., Ravichandran S., Dharmalingam S. (2024). Sulphonated graphene oxide as functionalized filler for polymer electrolyte membrane with enhanced anti-biofouling in microbial fuel cells. Colloids Surfaces A Physicochem. Eng. Asp..

[109] Liu Y., Zhou T., Yin G., Du K., Jia R., Yu X., Wang W., Guo J. (2025). Membrane fouling mitigation in Anoxic-Oxic membrane bioreactor with carbon nanotube/dopamine modification. J. Water Process Eng..

[110] Shen Y., Badireddy A.R. (2021). A Critical Review on Electric Field-Assisted Membrane Processes: Implications for Fouling Control, Water Recovery, and Future Prospects. Membranes.

[111] Li C., Guo X., Wang X., Fan S., Zhou Q., Shao H., Hu W., Li C., Tong L., Kumar R.R. (2018). Membrane fouling mitigation by coupling applied electric field in membrane system: Configuration, mechanism and performance. Electrochim. Acta.

[112] Dan Grossman A., Qi S., Aregawi Gebretsadkan A., Euni Beyioku O., Turkeltaub T., Shames A.I., Oren Y., Ronen A., Bernstein R. (2024). Mechanism of mitigating organic fouling on an electro-conductive membrane under anaerobic conditions and cathodic operation. Appl. Surf. Sci..

[113] Qian L., Yuan C., Wang X., Zhang H., Du L., Wei G., Chen S. (2023). Conductive MXene ultrafiltration membrane for improved antifouling ability and water quality under electrochemical assistance. RSC Adv..

[114] Ghosh U., Mukherjee S., Chakraborty S. (2021). Electrophoretic motion of a non-uniformly charged particle in a viscoelastic medium in thin electrical double layer limit. J. Fluid Mech..

[115] Hou B., Liu X., Zhang R., Li Y., Liu P., Lu J. (2022). Investigation and evaluation of membrane fouling in a microbial fuel cell-membrane bioreactor systems (MFC-MBR). Sci. Total Environ..

[116] Liu Y., Gao X., Cao X., Sakamaki T., Zhang C., Li X. (2022). Study on the performance and mechanism of bio-electrochemical system to mitigate membrane fouling in bioreactors. Bioresour. Technol..

[117] Anis S.F., Lalia B.S., Khair M., Hashaikeh R., Hilal N. (2021). Electro-ceramic self-cleaning membranes for biofouling control and prevention in water treatment. Chem. Eng. J..

[118] Xu X., Zhang H., Gao T., Wang Y., Teng J., Lu M. (2020). Customized thin and loose cake layer to mitigate membrane fouling in an electro-assisted anaerobic forward osmosis membrane bioreactor (AnOMEBR). Sci. Total Environ..

[119] Yang Y., Qiao S., Jin R., Zhou J., Quan X. (2019). Novel Anaerobic Electrochemical Membrane Bioreactor with a CNTs Hollow Fiber Membrane Cathode to Mitigate Membrane Fouling and Enhance Energy Recovery. Environ. Sci. Technol..

[120] Sapireddy V., Ragab A., Katuri K.P., Yu Y., Lai Z., Li E., Thoroddsen S.T., Saikaly P.E. (2019). Effect of specific cathode surface area on biofouling in an anaerobic electrochemical membrane bioreactor: Novel insights using high-speed video camera. J. Memb. Sci..

[121] Song J., Yin Y., Li Y., Gao Y., Liu Y. (2020). In-situ membrane fouling control by electrooxidation and microbial community in membrane electro-bioreactor treating aquaculture seawater. Bioresour. Technol..

[122] Du F., Hawari A., Baune M., Thöming J. (2009). Dielectrophoretically intensified cross-flow membrane filtration. J. Memb. Sci..

[123] Huotari H.M., Huisman I.H., Trägårdh G. (1999). Electrically enhanced crossflow membrane filtration of oily waste water using the membrane as a cathode. J. Memb. Sci..

[124] Bowen W.R., Kingdon R.S., Sabuni H.A.M. (1989). Electrically enhanced separation processes: The basis of in situ intermittent electrolytic membrane cleaning (IIEMC) and in situ electrolytic membrane restoration (IEMR). J. Memb. Sci..

[125] Porcelli N., Judd S. (2010). Chemical cleaning of potable water membranes: The cost benefit of optimisation. Water Res..

[126] Hou B., Zhang R., Liu X., Li Y., Liu P., Lu J. (2021). Study of membrane fouling mechanism during the phenol degradation in microbial fuel cell and membrane bioreactor coupling system. Bioresour. Technol..

[127] Halali M.A., Larocque M., de Lannoy C.-F. (2021). Investigating the stability of electrically conductive membranes. J. Memb. Sci..

[128] Zhang Z., Wang S., Hu W., Ma C., Cui C., Wang L. (2025). Mitigation of irreversible membrane biofouling by CNTs-PVDF conductive composite membrane. Environ. Res..

[129] Hu J., Cao X., Qu L., Khodseewong S., Zhang S., Sakamaki T., Li X. (2024). Study on the mechanism of mitigating membrane fouling in MFC-AnMBR coupling system treating sodium and magnesium ion-containing wastewater. Environ. Technol..

[130] Lei J., Yu F., Xie H., Ma J. (2023). Ti_3_C_2_T*_x_* MXene/carbon nanofiber multifunctional electrode for electrode ionization with antifouling activity. Chem. Sci..

[131] Zhu Y., Duan W., Huang Z., Tian L., Wu W., Dang Z., Feng C. (2024). An Anti-Scaling Strategy for Electrochemical Wastewater Treatment: Leveraging Tip-Enhanced Electric Fields. Environ. Sci. Technol..

[132] Zhang Y., Yan D., Zhao Y., Li J., Wang J., Wang Y., Wang J., Zhang H., Chen L., Zhang M. (2025). Pressure-induced piezoelectric response for mitigating membrane fouling in surface water treatment: Insights from continuous operation and biofouling characterization. Water Res..

[133] Alhajar A., Tawalbeh M., Arjomand D., Rahman N.A., Khan H., Al-Othman A., Kumar V., Kumar M. (2022). Chapter 14—Integrating forward osmosis into microbial fuel cells for wastewater treatment. Integrated Environmental Technologies for Wastewater Treatment and Sustainable Development.

